# Supplementary material for: Leprosy and lymphatic filariasis-related disability and psychosocial burden in northern Mozambique
Source: PLoS Negl Trop Dis. 2024 Aug 13;18(8):e0012342. doi: 10.1371/journal.pntd.0012342 (PMC11321553; doi:10.1371/journal.pntd.0012342)
Supplement: Attachment — Submitted filename: 240131 Morbidity leprosy-LF_plain.doc [file pntd.0012342.s001.doc]

# Leprosy and lymphatic filariasis-related disability and psychosocial burden in Northern Mozambique

Authors: Robin van Wijk*^1,2^, Litos Raimundo^3^, Domingos Nicala^3^, Yuki Stakteas^3^, Adelaide Cumbane^4^, Humberto Muquingue^4^, Julie Cliff^4^, Wim van Brakel^1^, Artur Muloliwa^5^.

1. NLR, Amsterdam, The Netherlands
2. Erasmus University Medical Center, Rotterdam, the Netherlands
3. NLR Mozambique, Maputo/Nampula, Mozambique
4. Universidade Eduardo Mondlane, Maputo, Mozambique
5. Universidade Lúrio, Nampula, Mozambique

## *Corresponding author: r.vanwijk@nlrinternational.org

## Abstract

**Introduction** Leprosy and lymphatic filariasis (LF) are among the most disabling neglected tropical diseases (NTDs) affecting the citizens of Mozambique, especially in the Northern provinces. The irreversible impairments caused by these NTDs often lead to psychosocial consequences including poor mental wellbeing, stigma and reduced social participation. Limited data on this is available for Mozambique, while this is urgently needed to better understand the true disease burden, and to support advocacy for scaling up interventions.

**Methods** A cross-sectional mixed-methods study was set-up. Mental distress was assessed with the Self Reporting Questionnaire (SRQ-20), participation restriction with the Participation Scale Short (PSS) and perceived stigma with the EMIC affected persons stigma scale (EMIC-AP). Additionally, semi-structured interviews were conducted with persons affected by leprosy or LF.

**Results** In total, 127 persons affected by leprosy and 184 persons affected by LF were included in the quantitative study component. Eight semi-structured interviews were conducted. In both disease groups, mental distress was found in ~70% of study participants, and ~80% of persons affected by leprosy and ~90% of persons affected by LF perceived stigma. Moderate to extreme participation restriction was found in ~43% of leprosy affected persons and in ~25% of the LF affected group. More severe disabilities were associated with higher scores for all questionnaires. Being part of a self-care group showed to have a positive impact on these outcomes. Persons affected by leprosy and LF felt excluded, dismissed as a person and experienced financial problems.

**Conclusion** The findings provide evidence that persons affected by leprosy and LF are not only confronted with physical impairments, but also experience significant disability in the psychosocial domain, including mental distress, participation restriction and stigma. These challenges need to be taken up urgently by NTD programmes to promote the inclusion and wellbeing of persons affected by NTDs.

Author Summary

Leprosy and lymphatic filariasis (LF) are severe tropical diseases affecting over 1 billion people, especially in poor regions. In Mozambique, where 11 neglected tropical diseases (NTDs) are common, there is limited information about the impact of leprosy and LF on people's mental and social well-being. Our study in Northern Mozambique, supported by NLR Mozambique and health authorities, aimed to understand this impact. We looked at mental distress, participation restriction, and stigma using surveys and interviews.

127 persons affected by leprosy and 184 persons affected by LF were included in the quantitative study component and eight semi-structured interviews were conducted.

Our findings showed that many people faced mental distress (70%), participation restrictions (33%), and health-related stigma (86%). The severity of leprosy and LF was linked to higher challenges. Our analysis revealed that disability, stigma, and marital status played a significant role. Self-care groups had a positive impact.

Despite some limitations, our study highlights the urgent need for better support. Addressing mental health, promoting inclusion, and adapting services to local needs are crucial. We recommend more research with diverse groups and suggest integrating mental health care into programmes for neglected tropical diseases. This study emphasises the importance of taking care of the overall well-being of those affected by leprosy and LF in Mozambique.

## Introduction

Leprosy and lymphatic filariasis (LF) are part of a diverse group of conditions called neglected tropical diseases (NTDs). [1] NTDs mostly affect people living in poor communities and have devastating consequences in terms of health, social and economic outcomes. It is estimated that over 1 billion people globally are affected by an NTD. In addition, the World Health Organization (WHO) estimates that NTDs cost around 200,000 lives and 19 million disability adjusted life years each year. [1] Forty percent of persons affected by NTDs live in Africa. [2]

Leprosy is one of the NTDs that disproportionately affects the poor. [3] It is caused by *Mycobacterium leprae* and manifests in the skin, eyes and peripheral nerves of the hands and feet and can cause permanent physical disabilities, stigmatisation and discrimination as well as and impaired mental health, which exacerbates the poverty of those affected. [3] Leprosy can lead to the loss of sensitivity and muscular function of the hands and feet, and blindness. The WHO divides leprosy-related impairments into three grades: Grade 0 means no impairments; Grade 1 means loss of sensation in hands or feet; and Grade 2 visible impairments. [4]

Lymphatic filariasis is an NTD caused by nematode parasites, which when they invade the body cause increasing difficulties in the lymphatic circulation, resulting in permanent changes in drained tissues often causing lymphedema or hydrocele and in worst cases, elephantiasis. [5] LF is considered one of the leading global causes of permanent or long-term disabilities. Lymphoedema and hydrocele severity can be graded as follows: Grade 0: No oedema; Grade 1: mostly pitting oedema; spontaneously reversible on elevation; Grade 2: mostly non-pitting oedema; not spontaneously reversible on elevation; Grade 3 (elephantiasis): gross increase in volume in a Grade 2 lymphoedema, with dermatosclerosis and papillomatous lesions. [6]

Leprosy and LF are among the most disabling NTDs affecting the people of Mozambique. Although data is scarce, it is known that Mozambique has been endemic for 11 NTDs since 1950, the diseases are mostly prevalent in the Northern provinces of the country. [7] Grau-Pujol et al. [8] summarised the presence of NTDs and found that much data is not available on a national level, and showed that extensive mapping of the incidence and prevalence of NTDs in Mozambique is needed. In 2021, Mozambique reported an incidence of 3,135 new leprosy cases of whom around 21% had Grade 2 disabilities at diagnosis. [9,10] Numbers for the incidence of lymphatic filariasis in the country are even more scarce. The WHO reported that 494,085 persons had received mass drug treatment for LF in 2021. [11] It is estimated that globally around 63% of persons affected by LF are men with hydrocele, and around 38% has lymphoedema of their lower limbs. Around 90% of persons who develop lymphoedema are left with chronic manifestations. [11]

The irreversible impairments caused by leprosy and LF often result in social disadvantage and potentially prevent affected persons from participating in key aspects of life, including work, family and social activities. [4,12] This is not only caused by the physical impairments, but it is also the result of multiple psychosocial factors, such as stigma, social exclusion and mental distress. These factors are associated with a lower quality of life and can contribute to aggravation of the disease itself, and the associated consequences. [13] Several studies have already shown the chronic consequences of leprosy and LF on mental wellbeing and social participation. [14–16] The WHO recognises that persons affected by NTDs and specifically leprosy and LF are at risk ~~for~~ of mental health conditions. The combination of the mental health burden and physical consequences of leprosy and LF have a negative health, social and economic impact. [17]

Although the evidence base for the associations between NTDs and disability, mental health, stigma and social exclusion is growing, data seems to be coming from a limited number of countries. [13,18] Until now, no data have been available on the levels of mental distress, participation restriction and stigma among persons affected by leprosy or LF in Mozambique. This information is urgently needed to better understand the disease burden in the country and its endemic regions, and to support advocacy for scaling up funding and implementation of available disease management, disability prevention and inclusion interventions. [19] The aim of this study is therefore to contribute to describing the psycho-social impact of leprosy and LF for persons affected who live in Northern Mozambique.

## Methods

### Study design and setting

This was a cross-sectional mixed-methods study to gain an understanding of levels of physical disability, mental distress, participation restriction, and perceived stigma of persons affected by leprosy or LF in Mozambique. The study was carried out in the Northern province of Nampula, in the rural areas of Erati and Memba districts. These districts are endemic for leprosy and LF. The latter are reported by local self-care groups but are often not officially registered by local authorities due to the lack of a rigorous surveillance system. The study was facilitated by NLR Mozambique and supported by the provincial health authorities. It focused on these districts because they are the most neglected. Members of the local community speak of high prevalence and no action by local authorities. Data collection took place from October 2020 to January 2021. The research was undertaken as part of the Coalition for Operation Research on Neglected Tropical Diseases (COR-NTD) funded project *Avaliação dos Serviços de Gestão da Morbidade, Prevenção da Deficiência e Inclusão para Pessoas Afetadas pela Lepra, Filaríase Linfática e Konzo.*

### Quantitative data collection

#### Sampling strategy and participants

The sample size of the quantitative component assumed a conservative prevalence of disability and psychosocial burden of 50% among persons affected by leprosy and LF, because we did not have actual data available. A lower or higher estimated burden would lead to a lower sample size. For the calculation, the EpiCalc2000 was used. [20] Parameters are prevalence 50%; precision of the 95%CI of +/- 10%. This gives a sample size of 96 as outcome. Based on this, we set a desired sample size of 100 participants per disease type.

Participants were approached by community volunteers who provide basic health services, basic clinical services, and spread information as part of the community health education they provide. Each volunteer gathered data in their own community and was therefore knowledgeable of where and how to recruit participants, and visited them at their homes to conduct the interviews, using the questionnaires. Through convenience sampling based on the knowledge of the community volunteers and snowballing participants were selected. Persons were included if they 1) were affected by leprosy or LF, 2) were living in one of the target districts (Erati or Memba) and, 3) consented to participate in the study. Persons were excluded from the study if they 1) were affected by more than one of the target diseases (leprosy **and** LF); 2) were diagnosed with another disabling condition or 3) were unable to understand the study, its advantages or risks.

#### Data collection

Electronic data collected included socio-demographic, health services and disability data.

Three standardised questionnaires were used to assess the levels of mental distress, participation restriction and experienced stigma, respectively the Self Reporting Questionnaire consisting of 20 questions (SRQ-20), Participation Scale Short with 13 questions (P-scale Short), and the 15-question EMIC affected persons stigma scale (EMIC-AP). [21–23] The community volunteers received a two-day training which included grading disability and lymphoedema, conducting the interviews using questionnaires and data entry into a mobile application via tablets.

The questionnaires were first forward translated by two professional translators into the local language “Makhuwa”. Second, they were translated back to English by a third professional translator without any prior knowledge of the questionnaires. Based on the backtranslation, several alterations were made to the Makhuwa versions of the questionnaire. Third, the questionnaires were piloted by the volunteers who would also gather the data in the two study districts. Based on their findings, for each questionnaire two final versions were developed, one for each district due to differences in dialects.

The full data collection was done electronically on a tablet and the mobile application from data collection software REDCap. [24]

**Socio-demographic, health services and disability data**

The full interview started with several questions regarding socio-demographics, such as age, sex and civil status. Questions about the available health services included the distance in terms of time to reach the nearest health facility, how participants would travel there and whether they were included in a self-care group. Disease-specific disability data concerned the disability grade and whether the participant needed assistive devices for mobility.

**SRQ-20**

The Self-Reporting Questionnaire (SRQ-20) was developed to measure mental distress, and includes the main signs and symptoms of mental health problems [21,25]. The tool was especially developed for people living in low- and middle-income countries (LMICs) and is easy to use. The SRQ is a screening instrument and consists of 20 questions, which are answered by ‘yes’ or ‘no’. Questions ask about physical as well as psychological symptoms of mental distress. When a symptom was present in the last month the question is scored with a 1, while a score of 0 indicates the symptom was absent. Therefore, the highest possible score is 20, and a high total score indicates the presence of mental distress.

**Participation Scale Short (P-scale Short)**

The P-scale Short measures respondent-perceived restrictions in social participation. The tool is a 13-item questionnaire based on the terminology and conceptual framework of the WHO International Classification of Functioning (ICF). [22] The P-scale Short is a shortened version of the original Participation scale, which consists of 18 items. The severity of the restrictions in work and social participation is measured quantitatively. The questions are based on all the key domains that represent the ICF component of Participation, such as work, family and community involvement. It asks the participant to compare their level of participation to that of a peer and then to assess their level of restriction on this topic. It is easy to use, as it includes yes/no questions and a 1-5 response (likert) scale for each item. The participation score is the sum of the ratings of all items, with a minimum score of 0 (no participation restriction) and a maximum score of 65 (severe participation restriction). The P-scale has proven to be valid for use in different cultural environments and was found suitable to be conducted by non-professional interviewers. [23]

**Explanatory Model Interview Catalogue Stigma Scale for affected persons (EMIC-AP)**

The Explanatory Model Interview Catalogue (EMIC) is based on the framework of cultural epidemiology, and has been developed to reflect cultural perceptions, beliefs and practices related to illness. [26] The EMIC Stigma Scale for affected persons is derived from the EMIC and measures anticipated, experienced and internalised stigma related to health conditions. It covers certain aspects of life that can be affected by stigma, such as disclosure, pity, shame, avoidance, respect, being made fun of and marriage prospects. [26] It consists of a list of items that probe stigma perception and experience by the person affected. [27] The EMIC consists of 15 items with possible scores between 0 and 3 for each item. The total score ranges from 0 to 45. The International Federation of Anti-Leprosy Associations (ILEP), the stigma research workshop held in Amsterdam in 2010 and the ILEP/NNN (Neglected Tropical Disease NGO Network) Stigma guides classified the EMIC-AP stigma scale as one of the recommended instruments to measure perceived stigma in persons affected by leprosy. [28]

### Qualitative data collection

Semi-structured interviews were conducted with persons affected by leprosy or LF, community leaders and a representative of a disabled people’s organisation (DPO). Data collection took place in July and August 2020. The interviews focused on the healthcare resources available and their needs for Morbidity Management and Disability Prevention (MMDP)/Disease Management and Disability Inclusion (DMDI) services, and were based on the Community Health Assessment and Group Evaluation (CHANGE) Action Guide by the Centers for Disease Control and Prevention, Atlanta. [29] MMDP is a broad strategy involving both secondary and tertiary prevention. [30] Secondary prevention includes simple hygiene measures, such as basic skin care. DMDI is similar to MMDP but is a wider, more holistic concept. [31] DMDI also comprises tertiary prevention that includes psychological and socioeconomic support for people with disabling conditions to ensure that they have equal access to rehabilitation services and opportunities for health, education, and income. Follow-up questions were asked based on the experiences shared by the participants and often also included aspects of mental well-being and social participation. The interviews were conducted in the commonly spoken *Makhuwa* language.

The participants were selected through convenience sampling, and at times by snowball sampling. In each district, contact persons of the governmental health system (district focal points) were informed about the date of the arrival of the research team. They then made appointments with the community leaders and affected persons, based on their availability. Participants were eligible for inclusion if they 1) were affected by leprosy or LF; or were a community leader; or were a member of the disabled people’s organisation ADEMO, 2) were living in one of the target districts (Erati or Memba) and 3) consented to participate in the study. Persons were excluded from the study if they were unable to understand the study, its advantages or risks.

### Data analysis

#### Quantitative

Before analysis, the data was exported from the REDCap database to Excel and was thoroughly reviewed and cleaned: records were matched to the paper-based informed consent forms, duplications were deleted, and records were checked to match with the in- and exclusion criteria. Subsequently, the records were anonymised.

Data was analysed by disease group and also aggregated for the total group of participants. Socio-demographic, health services and disability data were analysed using descriptive statistics and expressed in means, medians, frequencies and percentages. Analysis was performed in SPSS v27.

The outcome of each questionnaire determined the level of one of the domains that is researched in this study. For both diseases, data was collected using the SRQ-20 for the level of mental distress, the P-scale Short for level of participation restriction, and the EMIC-AP for the level of anticipated and experienced stigma. Missing values were imputed by calculating the study participant specific average score on the scale. If more than two values for one scale were missing, the participant was excluded from the total score calculations.

Cut-off points were adopted from previous studies that used the SRQ-20 and P-scale Short to investigate leprosy. For the SRQ-20, a score ≥8 implies probable mental distress. [32] For the P-scale Short, categories are: 0-6 indicates no significant restriction, 7-13 indicates a mild restriction, 14-30 indicates a moderate restriction, 31-50 severe restriction and 51-65 extreme restriction. [33] For the EMIC-AP, no cut-off point can be objectively measured, since it cannot be used with unaffected community members. However, in line with the approach taken by Sermrittirong et al., the cut-off point of ≥8 was chosen. [34] This is based on the consideration that participants can be said to perceive stigma when they answered at least four questions with ‘yes’, or eight questions with ‘possibly’ at least, or a mixture of these.

To test the internal consistency of the questionnaires, Cronbach’s alpha was calculated. In addition, the outcomes of the three scales were compared between several sociodemographic subgroups. For normally distributed data, a two-sample t-test was used to test for a difference between two groups. To detect significant differences between multiple groups, one-way analysis of variance (ANOVA) was used and complemented by the post-hoc Tukey procedure. When data was not normally distributed, a Kruskal-Wallis test was used to test the significance of differences of median scores. This test detects rank-based differences between the groups and determines whether these are significant. When a significant difference was detected, the pair-wise Mann-Whitney test was used to determine the differences between each pair of groups within the variables.

A multivariate analysis was carried out using a model with all demographic variables potentially associated with the outcome, with a p-value of <0.2 identified through univariate analysis. Variables with p-values ≥0.05 were eliminated one by one until all variables that remained in the model were statistically significant (p<0.05). The non-normally distributed data was bootstrapped as part of the regression procedure when adding it into the multivariate model.

#### Qualitative

Audio-recorded data was translated and transcribed into Portuguese. A unique identifying number was assigned to each participant to ensure anonymity. Data was subsequently coded with the use of Atlas.ti version 8. Coding was done inductively: Open coding took place; codes were derived from the data. In other words, coding was performed through an inductive process. During analysis, a coding scheme was developed. Findings in interviews that validated or contradicted one another were matched to create data triangulation. Findings were discussed with the native Makhuwa and Portuguese-speaking researchers and agreement was reached on the included results, the interpretation and translation.

### Ethical considerations

Permission from the *Comité Nacional de Bioética para a Saúde* was obtained to carry out this study. Prior to their inclusion, participants were informed of the research aims, the voluntary basis of their participation and their rights. They signed, or thumb printed, paper-based informed consent forms.

## Results

In total, 315 participants were included in the quantitative component of the study. During the data cleaning process four persons were excluded because they had a double disease or disability burden: three were diagnosed with two of the studied diseases, the fourth person was also diagnosed with polio.

Table 1 shows the demographics of the included research population of the quantitative study arm in total and per disease group. In total, 127 persons affected by leprosy and 184 persons affected by LF were included in the analysis. The mean age of the study population was 50. Of the 127 participants affected by leprosy, 56 (44%) were women. The mean age of participants affected by leprosy was 49 years. Fifty-three (42%) were recruited in Erati and 74 (58%) in Memba district. Fifty-two percent of the persons affected by leprosy were married, and 40% single.

Of the 184 persons affected by LF, 61 were female (33%). The mean age of persons affected by LF was 52. Seventy-six (41%) of them were recruited in Erati, 108 (59%) were recruited in Memba. Seventy-seven percent were married and 17% was single.

In both districts, participants indicated the need to travel for a long time when they needed to visit a health unit, this was about 1-2 hours in Erati and 2 hours or more for people living in Memba. However, in Erati, 94% indicated to have to go by foot, while in Memba 39% also mentioned the option to use a (motor)bike. Almost 80% of the participants recruited in Erati included persons affected by leprosy with a Grade 2 disability, whereas this was around 50% for Memba. For LF, a more comparable rate of persons with severe lymphoedema was found in both districts: in Erati 53% had Grade 2 or Grade 3 lymphoedema and in Memba this was 57%. In Erati, 19% of the included persons affected by leprosy and 15% by LF were included in self-care groups. In Memba this was 11% and 1% respectively.

Table 1: Study population characteristics – quantitative study

|  |  | **Leprosy** | | **LF** | | **Total** | |
| --- | --- | --- | --- | --- | --- | --- | --- |
|  |  | n | % | n | % | n | % |
| **Total included** |  | 127 |  | 184 |  | 311 | 100 |
| **Sex** | Male  Female  Unknown | 70  56  1 | 55.1  44.1  0.8 | 122  61  1 | 66.8  33.2  0.5 | 192  117  2 | 61.7  37.6  0.6 |
| **Age (years)** | Mean (SD)  Unknown | 49.2 (16.8)  1 | 0.8 | 51.6 (14.3)  5 | 2.7 | 50.6  (15.4)  6 | 1.9 |
| **District of residence** | Erati  Memba  Unknown | 53  74  0 | 41.7  58.3 | 76  108  1 | 41.3  58.7  0.5 | 129  182  1 | 41.5  58.5  0.3 |
| **Marital status** | Single  Married*  Divorced  Widowed | 51  66  3  7 | 40.2  52.0  2.4  5.5 | 32  142  3  7 | 17.4  77.2  1.6  3.8 | 83  208  6  14 | 26.7  66.9  1.9  4.5 |
| **Disability grade** | 0  1  2  3  Unknown | 18  31  78  n/a | 14.2  24.4  61.4  n/a | 71  71  29  13 | 38.6  38.6  15.8  7.1 | n/a  n/a  n/a  n/a | n/a  n/a  n/a  n/a |
| **Included in self-care group** | Yes  No | 18  109 | 14.2  85.8 | 12  172 | 6.5  93.5 | 30  281 | 9.6  90.4 |

*Married or co-habitation

In the results from the qualitative study, findings were derived from the five interviews with persons affected by leprosy or LF. In addition, two interviews with community leaders were analysed in this context. Finally, an interview with a representative from ADEMO was analysed. ADEMO is a DPO active in several district of Nampula, among which the studied districts. Table 2 shows the characteristics of the eight interviewees included in the study. In total, two women and six men were interviewed. Their ages ranged from 21 to 84 years old. They are all farmers, living on subsistence agriculture, although some of them were impeded by their disabilities.

Table 2: Study population characteristics – qualitative study

| **Participant** | **Sex** | **Age** | **Civil status** | **Education level** | **Role** | **District** |
| --- | --- | --- | --- | --- | --- | --- |
| **1** | M | 50 | Single | Primary | Community leader | Memba |
| **2** | M | 56 | Married | Primary | Person affected by leprosy | Memba |
| **3** | M | 21 | Single | Primary | Person affected by leprosy | Memba |
| **4** | F | 55 | Married | Primary | Person affected by LF | Memba |
| **5** | F | 46 | Married | Primary | Community leader | Erati |
| **6** | M | 64 | Married | Primary | Person affected by LF | Erati |
| **7** | M | 84 | Married | None | Person affected by leprosy | Erati |
| **8** | M | 46 | Single | Primary | ADEMO representative | Erati |

### Disease morbidity and disability

In the quantitative study component, the majority of participants affected by leprosy had Grade 2 disabilities (61%), 24% had Grade 1 disabilities and 14% did not have disabilities (Grade 0). Out of the persons affected by leprosy, eighteen (14%) were part of a self-care group.

Grade 1 Grade 2The majority of persons affected by LF had Grade 1 lymphoedema (39%), closely followed by 39% of the participants with Grade 2 lymphoedema. 16% had grade 3 lymphoedema, and for 7% it was not graded. Out of the 122 men affected by LF included in the study, 96 had hydrocele (79%) and 7 had both lower limb lymphoedema and hydrocele (6%). Among the persons affected by LF, 7% was part of a self-care group.

Disabilities reported in the interviews by the affected persons were visible in their hands and legs. Persons affected by leprosy with disabilities in their legs reported pain, tingling, burning sensations and itching as well as loss of strength. These all led to a decreased walking ability. Both respondents affected by LF said to experience severe pains in their legs, which impedes walking. They also reported periods of inflammation during which the pain and swelling become more severe.

“*There are rehabilitation services for those that fall and break their leg for example, but I have never seen people that have LF went there*” – Person affected by LF, Memba

The representative from ADEMO explained that people with disabilities in general are regularly excluded from the community. He himself was left by his parents because of a disability in his leg. He mentioned regular name-calling of persons with disabilities. He also said that persons with disabilities are told that they cannot go to school, and when they do, they are scolded for everything that they do wrong. People with disabilities have difficulties getting married, or people are divorcing them.

|  |  | **Leprosy** | **LF** | **Total** |
| --- | --- | --- | --- | --- |
| **SRQ-20** | N  Mean (95% CI)  % above cut-off point (≥8) | 126  9.89 (9.0-10.8)  68.5% | 182  10.0 (9.3-10.7)  70.1% | 308  9.97 (9.4-10.5)  69.5% |
| **P-scale Short** | N  Median (IQR)  no restriction  Mild  Moderate  Severe  Extreme | 126  10 (2-32.5)  43.3%  12.6%  16.5%  20.5%  6.3% | 184  5.5 (0-14.8)  55.4%  19%  15.2%  8.7%  1.6% | 310  6 (0-20)  43.3%  12.6%  20.5%  26%  8% |
| **EMIC-AP** | N  Mean  95% CI  % above cut-off point (≥8) | 127  20.3 (18.2-22.3)  79.5% | 184  20.5 (19.0-22.0)  89.7% | 311  20.4 (19.2-21.6)  85.5% |
| CI: confidence interval; IQR: inter-quartile range | | | | |

*Table 3: Scores per questionnaires and disease type*

Outcomes of the three standard questionnaires are recorded in Table 3, outcomes by sociodemographic sub-group can be found in Tables 4 and 5. The percentage of affirmative answers for mental distress (SRQ-20) can be found in figure 1, for participation restriction (PSS) in figure 3, and for stigma (EMIC) in figure 4.

### Mental distress

Internal consistency of the SRQ-20 was good for the total sample (α=0.83), and for the disease samples separately, leprosy: α=0.85, LF α=0.81. Of the total sample, 70% experienced mental distress.

Figure 1 - SRQ-20 percentage of affirmative answers per disease

Based on the SRQ-20 cut-off value, 69% of persons affected by leprosy experienced mental distress. Significant differences were found between the three disability grade groups, with a higher mental distress score for the higher disability grades (*p*<0.001). Using the same cut-off value, 70% of persons affected by LF experienced mental distress. We found significantly different mental distress scores between persons with different lymphedema grades, however no clear correlation between mental distress and lymphedema severity could be detected. An overview of this can be found in figure 2.

Figure 2 - Mental distress, participation restriction and stigma scores per disability/lymphedema grade

### Participation restriction

Internal consistency of the P-scale Short was good for the total sample (α=0.92), and for the disease samples separately, α=0.93, LF α=0.89. Of the total population, 33% experienced moderate or worse participation restriction.

Based on the P-scale Short cut-off value, 43% of persons affected by leprosy experienced a moderate or worse participation restriction. A significant difference in participation restriction was found for persons affected by leprosy who were married (median=5 IQR=0-13.25) compared to those who were single (median=20.5 IQR=2.75-40) (*p*=0.002). Persons affected by leprosy reported restrictions most frequently on question 1 and 2 (work participation): “Do you have equal opportunity as your peers to find work?” and “Do you work as hard as you peers do?”

One of the persons affected by leprosy explained that the availability of assistive devices could help him to get around more: “*It would be good if there was something that could help me move, maybe a bicycle, then I could reach the health unit and go home more quickly.*” – Person affected by leprosy, Memba.

Figure 3 - Percentage of answers confirming a participation restriction per disease

Similarly, 26% of persons affected by LF experienced a moderate or worse participation restriction. Persons with grade 3 (median=15 IQR=9.5-30) lymphoedema experienced significantly worse participation restriction than persons with lower grades of lymphoedema. Persons affected by LF reported the highest level of restriction on question 1 and 2: “Do you have equal opportunity as your peers to find work?” and “Do you work as hard as you peers do?”

Both respondents affected by LF said to experience severe pains in their legs, which impedes walking. They also reported periods of inflammation during which the pain and swelling become more severe, during this time they had no other option to stay at home and depend on others for water and food, they were not able to work. “*… I can't work, I'm a man who should not be working anymore, but I cannot afford to stop … Money is a real problem … To have food or not to have food*”– Person affected by LF, Erati.

Table 4: Leprosy: SRQ-20, PSS and EMIC-AP scores per sociodemographic outcome

|  |  |  | **SRQ-20** | **PSS** | **EMIC** |
| --- | --- | --- | --- | --- | --- |
|  |  | **n** | **Mean**  **(95%CI)** | **Median (IQR)** | **Mean**  **(95%CI)** |
| **Sex** |  |  | *p=0.466* | *P=0.344* | *p=0.747* |
|  | **Male** | 70 | 9.5  (8.5-10.7) | 10  (0-33.5) | 20.4  (17.8-23.2) |
|  | **Female** | 56 | 10.2  (8.8-11.7) | 11  (3-33) | 19.8  (16.7-23.0) |
| **Age** |  |  | *p=0.965* | *p=0.053* | *p=0.447* |
|  | Correlation coefficient | Mean  =49 | Pearson: -0.123 | Spearman: 0.174 | Pearson: -0.196 |
| **Marital status** |  |  | *p=0.152* | ***p<0.001*** | *p=0.026* |
|  | **Married*** | 66 | 9.9  (8.7-11.0) | 5 (0-13.25) | 21  (18.2-23.8) |
|  | **Unmarried** | 61 | 9.9  (8.5-11.2) | 27  (4-44.5) | 19.4  (16.4-22.4) |
| **Included in self-care group** |  |  | *p=0.280* | ***p=0.005*** | *0.182* |
|  | **Yes** | 18 | 11.1 (9.4-12.6) | 3 (0-11.25) | 23.6 (18.5-28.7) |
|  | **No** | 109 | 9.7 (8.7-10.7) | 11.5 (2.55-38.75) | 19.7 (17.4-21.9) |
| **Disability grade** |  |  | ***p<0.001*** | *p=0.105* | ***p<0.001*** |
|  | **G0** | 18 | 5.2  (3.0-7.4) | 6  (2-22.25) | 8.6  (5.7-11.5) |
|  | **G1** | 31 | 8.8  (7.1-10.6) | 7  (3-17.33) | 17.0  (12.9-21.1) |
|  | **G2** | 78 | 11.4  (10.4-12.4) | 15  (1-40) | 24.2  (21.9-26.6) |

*Married or co-habitation; **Bold** = significant after Bonferroni correction - p-values <0.0025; CI = confidence interval; IQR= interquartile range

Table 5: Lymphatic Filariasis: SRQ-20, PSS and EMIC-AP scores per sociodemographic outcome

|  |  |  | **SRQ-20** | **PSS** | **EMIC** |
| --- | --- | --- | --- | --- | --- |
|  |  | **n** | **Mean**  **(95%CI)** | **Median (IQR)** | **Mean**  **(95%CI)** |
| **Sex** |  |  | *p=0.005* | *p=0.012* | *p=0.682* |
|  | **Male** | 122 | 9.3  (8.5-10.6) | 5 (0-12) | 20.3  (18.4-22.1) |
|  | **Female** | 61 | 11.3  (10.3-12.4) | 9 (2.5-19.5) | 20.9  (18.2-23.6) |
| **Age** |  |  | *p=0.345* | ***p=0.015*** | *p=0.631* |
|  | Correlation coefficient | *Mean=*  *52* | Pearson: 0.071 | Spearman: 0.182 | Pearson: -0.036 |
| **Marital status** |  |  | *p=0.636* | ***P<0.001*** | *p=0.703* |
|  | **Married*** | 142 | 10.1  (9.4-10.8) | 5 (0-11) | 20.6  (19.1-22.3) |
|  | **Unmarried** | 42 | 9.7  (8.1-11.4) | 11.5 (3.8-36.5) | 20.0  (16.2-23.8) |
| **Included in self-care group** |  |  | *p=0.044* | *p=0.374* | ***p=0.004*** |
|  | **Yes** | 12 | 12.6 (10.9-14.2) | 9 (2.75-21.5) | 28.7 (22.9-32.4) |
|  | **No** | 172 | 9.8 (9.1-10.5) | 5 (0-14.75) | 20.0 (18.4-21.5) |
| **Lymphedema grade** |  |  | ***p<0.001*** | ***P=0.002*** | ***P=0.007*** |
|  | **G1** | 71 | 11.8 (11.0-12.7) | 4 (0-12) | 23.9 (21.5-26.3) |
|  | **G2** | 71 | 8.6 (7.6-9.8) | 5 (0-9) | 18.7 (16.4-21.0) |
|  | **G3** | 29 | 9.6 (8.0-11.1) | 15 (9.5-30) | 21.1 (17.8-24.5) |

*Married or co-habitation; **Bold** = significant after Bonferroni correction - p-values <0.0025; CI = confidence interval; IQR= interquartile range

### Stigma

Internal consistency of the EMIC-AP was good for the total population (α=0.82), and for the disease samples separately, leprosy: α=0.84, LF α=0.81. Of the total population, 86% experienced health-related stigma.

One of the community leaders mentioned that leprosy was caused by a spell. He also explained that he was not in favour of letting people affected by leprosy travel to health units, because on their way there they may infect many others. His beliefs about this had been reinforced by the health services that set up groups for persons affected by leprosy in the community; he thought these were a way to prevent persons affected from travelling to the health units.

**Leprosy**

The outcomes of the EMIC-AP scale showed that 80% of persons affected by leprosy experienced health-related stigma. A significant difference in experienced health-related stigma was found for persons affected by leprosy with disability Grade 2 compared to disability Grade 0 and Grade 1. The difference in EMIC-AP outcomes was also significant between Grade 0 and Grade 1 disability (p=0.016).

A person affected by leprosy was told that he should stay at home and needed to be treated with traditional medicine. Another was told that leprosy was not curable and that he needed to be isolated in a place outside of the community. He also explained that he should not sleep next to others or touch others, because they may also get the disease. He explained that he should regularly wash, because the smell of his sweat could contaminate others. Another person affected by leprosy said that some people are born with leprosy, that they get infected in their mother’s womb.

Exclusion was mostly mentioned in the context of leprosy. Both community leaders and the representative of ADEMO specifically mentioned that persons affected by leprosy were regularly excluded from the community because of fear of transmission. They had heard from people affected by leprosy living apart from the rest and lacking support by family and friends. Persons affected by leprosy confirmed this. One of them explained that he no longer could live with his parents:

“*My mother and father despised me . . . I built my own house where I sleep alone. In this house, I sleep alone and I keep it closed.*” – Person affected by leprosy, Memba.

Figure 4 - EMIC Percentage of affirmative answers indicating stigma per disease

**Lymphatic filariasis**

According to the outcomes of the EMIC-AP questionnaire, 90% of persons affected by LF experienced health-related stigma. A significant difference was found between persons with Grade 1 and Grade 2 lymphoedema (p=0.009). The persons with Grade 1 lymphoedema experienced a higher level of health-related stigma compared to those with Grade 2 lymphoedema. Other than these significant differences, no correlation between experienced stigma and lymphedema severity was found in our sample. Persons affected by LF reported highest scores on item 1 and 3: “If possible, would you prefer to keep people from knowing about your disease?” and “Do you think less of yourself because of your disease?”.

A man affected by LF explained that his family members viewed him as an old man that cannot do anything after he experienced an attack of inflammation. *“…the whole family knows that “our old man here is sick*” – Person affected by LF, Erati.

Table 6: Multivariate analysis

|  | **SRQ** | | | **PSS** | | | **EMIC** | | |
| --- | --- | --- | --- | --- | --- | --- | --- | --- | --- |
|  | Model | B (p-value) | R^2^ | Model | B (p-value) | R^2^ | Model | B (p-value) | R^2^ |
|  | Disability (yes/no) | 1.728 (p=0.09) | 0.475 | Married (yes/no) | 13.62 (p=0.001) | 0.219 | Age | -0.080 (p=0.070) | 0.514 |
| Leprosy | Stigma (EMIC) | 0.271 (p<0.001) |  | Disability (no-mild/severe) | 7.981 (p=0.016) |  | Disability (no-mild/severe) | 5.121 (p=0.002) |  |
|  |  |  |  | Self-care group | 9.467 (p=0.004) |  | Mental distress (SRQ) | 1.342 (p<0.001) |  |
|  |  |  |  |  |  |  |  |  |  |
| LF | Sex | 1.669 (p=0.004) | 0.362 | Age | 0.251 (p=0.001) | 0.462 | Self-care group | -5.389 (p=0.039) | 0.314 |
|  | Lymphoedema (mild/severe) | 1.652 (p=0.003) |  | Married (yes/no) | 10.42 (p=0.002) |  |  |  |  |
|  | Stigma (EMIC) | 0.226 (p<0.001) |  | Stigma (EMIC) | 0.245 (p=0.015) |  | Mental distress (SRQ) | 1.190 (p<0.001) |  |

The multivariate models are displayed in Table 6. The results for the SRQ show that for persons affected by leprosy, having a disability and experienced stigma remained significant in the multivariate analysis, accounting for 48% of the variability. For LF, 36% of the variability was explained by the female sex, a severe lymphoedema grade and stigma.

For persons affected by leprosy, 22% of variance in participation restriction was explained by marital status, with persons who were not married experienced more severe restrictions, by a more severe disability grade and not being in a self-care group. For persons affected by LF 46% of the variance in participation restriction could be explained by age, marital status and experienced stigma.

For persons affected by leprosy, higher levels of stigma were found for those who were younger, experiencing more severe disabilities and those with higher mental distress explaining 52% of the variability. For persons with LF only being part of a self-care group and having higher mental distress remained significant in the multivariate model.

## Discussion

The findings provide evidence that persons affected by leprosy and LF are not only confronted with physical impairments, but also experience their disability in the psychosocial domain, including mental distress, participation restriction and stigma. This supports the statement that individual characteristics and environmental factors interact to produce or ameliorate the dimensions of disability [35,36]. The questionnaires used exhibited high internal consistency in both disease groups, indicating that answers were consistent across the questions, thus likely to measure the same concept or construct. Mental distress was evident in a substantial proportion of the study population, while participation restriction was encountered in half of the study sample for both persons affected by leprosy and LF. A high level of perceived and experienced stigma was found in both disease groups and was encountered in two-thirds of the participants in the total study population. The existence of stigma towards persons affected by leprosy and LF was widely acknowledged and emphasised during the interviews.

The current study findings show that higher disability grades, or lymphoedema or hydrocele severity grades correlate with higher scores on all three scales, this should be taken into account when interpreting and comparing the results with other studies, as well as when planning interventions aimed to reduce mental distress, stigma and participation restrictions. Over half of the persons affected by leprosy included in this study had Grade 2 disabilities (61%), The national average among new cases in 2019 and 2020 was just over 19%. [37] A recently published study on the leprosy incidence of three neighbouring districts to our study districts shows a Grade 2 disability rate of 2 to 14% at the time of diagnosis. [38] This shows that our sample was strongly biased towards persons with more severe disabilities. This is understandable given convenience and snowball sampling used, which is likely to lead to persons with visible signs. While this still illustrates the severe impact of leprosy in this group, it limits the generalisability of the findings. In addition, in the sample of persons affected by LF, 79% of the men (53% of the total sample) had hydrocele. 67% of the included persons affected by LF were male. Given the global and expected distribution is that 63% of persons affected have hydrocele, the current study sample may have a slight underrepresentation of hydrocele cases. [11] Lower limb lymphoedema may be more difficult to hide and lead to other activity limitations than hydrocele does. It should therefore be considered that the sample was somewhat biased towards persons with lower limb lymphoedema.

The high levels of mental distress encountered in the study sample could consistently be related to the stigma experienced by the persons affected by both leprosy and LF. For leprosy, a clear relationship with the level of disability was also found. Adekeye et al. described how these stressors influence the well-being of persons affected by NTDs. [39] They explain how these stressors affect their livelihoods and how these results “*in a heightened sense of anxiety or worry among affected populations*” (i107). This mechanism could very well also explain the high mental distress in our population.

Many persons affected by leprosy and LF reported moderate or severe participation restrictions. As expected, those with more severe disabilities seemed most restricted. It is important to consider that it is not only physical impairment that restricts them from taking part in important roles in life; also the impact of stigma and mental distress plays an important role. The restrictions were especially experienced in their ability to work. Van ‘t Noordende et al. reported similar experiences of persons affected by leprosy and non-filarial and filarial lymphoedema, indicating that they especially had problems working the same way as they did before. [40]

Persons affected by leprosy reported high levels of anticipated and experienced stigma, this was worse for those with visible disabilities (Grade 2). Persons affected by leprosy would prefer not to disclose their disease status, and generally thought less of themselves. Leprosy is known for its high levels of stigma. [41] Like in the current study, studies from Nepal and Brazil, that used the EMIC-AP, reported that the highest percentage of affirmative answers was also given to the questionnaire items about disclosure and self-depreciation. [42,43] A qualitative study by Ebenso et al. from Nigeria about the stigmatisation of leprosy narrated the way persons affected by leprosy often feel no other option than to conceal their disease status, which leads to treatment avoidance and increasing the severity of disabilities. [44] In the current research, higher perceived stigma scores were associated with more severe disability grades, indicating that this cycle of stigmatisation may also exist in the study population in Mozambique. Very high levels of perceived stigma were also found among persons affected by LF. Two other studies from Nigeria mentioned persons affected by LF to have dropped out of school, having difficulties in their marriage, and loss of income. [45,46] Hofstraat & van Brakel performed a systematic review on stigma and NTDs and reported that “*Medium to high levels of stigma were found by the 18 studies that cover stigma related to LF*” (p.i56), whereas leprosy-related stigma was described as “severe”. [13] In the current study, both leprosy and LF affected persons reported extremely high levels of stigma, with a similar mean, but the percentage above the cut-off point was 10% higher in the LF group. So far very little evidence has been published about stigma and NTDs in Mozambique, and therefore the current study findings cannot be compared to other similar studies among the Mozambican population. One of the explanations for the high numbers of reported stigma could be related to the high percentage of persons with (severe) disabilities in the current sample.

Around 70% of the persons affected by leprosy and LF experienced mental distress. They had difficulties with thinking clearly and indicated that their daily work was suffering. More severe disability and lymphoedema grades were related to higher levels of distress. Mental distress was also slightly higher for women affected by LF compared to men. A study by Dorst et al. from Nepal about the mental wellbeing of persons affected by leprosy showed poor mental wellbeing in 38% of the participants and about 50% of the study participants had thoughts about suicide. [47] These findings were also associated with higher disability grades. [47] Two qualitative studies from the same country explained that mental wellbeing was mostly impacted by a lack of support from family and other social networks. [48,49] Obindo et al. who also focused on persons affected by leprosy, found psychiatric morbidity in about 38% of the study population. [50] Studies showed a significant impact of LF on mental wellbeing. The same researchers in Nigeria found that 20% suffered from depression, and that 74% of the study participants had a low self-esteem. [50]. Abdulmalik et al. narrated that persons affected by LF felt unhappy, sad, were crying a lot, felt worried and had suicidal thoughts. [51] Findings are in line with the results from the current study, where feelings of unhappiness were frequently expressed. In addition, Lund et al. showed that the difficulties with finding and retaining work were associated with poorer mental wellbeing. [52] The SRQ-20 that was used in the current study screens for mental distress, which is different from identifying depression. Higher percentages of mental distress do not directly indicate psychiatric illness but do show that mental wellbeing is being negatively affected.

In this study, it appears that mental distress and stigma can mutually influence each other. In any case, it is unlikely that there is only one pathway. In the literature, an overlap is reported between signs of mental distress and signs of stigma, such as feelings of guilt, decreased feelings of self-worth and withdrawal and isolation. [53–55] Experiencing stigma could therefore trigger or worsen mental distress, although the way someone feels mentally could also affect the way they perceive stigma. Sharaf et al. and Oexle et al. have indicated that, even when accounting for the influence of depression, people experiencing stigma may still encounter suicidal thoughts. [56,57] Carpiniello et al. found that suicidal ideation can also stem from a significant decrease in self-esteem and a sense of hopelessness. [58] These experiences can also occur in people who experience stigma. This will need to be considered when providing mental health support to persons affected by NTDs, to also focus on experienced stigma.

Although only 14% of leprosy-affected persons and 7% of LF-affected persons were included in a self-care group, the results show a clear positive impact of these groups on the psychosocial wellbeing of persons affected by leprosy or LF. Deepak et al. conducted qualitative research among leprosy self-care groups in the same province in Mozambique and reported that socialising, better care for disabilities and wounds, and fighting for their rights were among the main benefits for participants of these groups. [59] They argued that “*self-care activities can have a fundamental role in the prevention of progressive worsening of leprosy-related impairments*” (Deepak et al., p.290). This was also published in a country profile for leprosy in Mozambique, stating that “*the level of stigma in the community is decreasing, at least in the areas where self-care groups and DPOs* [disabled peoples organisations] *are active*” (De Kruijff, p.92). [60] In addition to the physical benefits of being involved in self-care group activities, our results show that the psychosocial benefits for persons affected by NTDs should not be underestimated.

**Limitations**

The current study had several limitations. One of these is the sampling of study participants. As the recruitment of study participants was done mainly by community volunteers, they were more likely to include persons affected by leprosy and LF who were known to be affected and therefore have visible disabilities. This means that persons with less severe disabilities or lymphoedema/hydrocele or who were concealing their condition, were likely to be overlooked. This has led to a study sample which is not representative of the entire study population. However, it does show the high impact of physical disabilities on mental wellbeing, social participation, and stigma.

A second limitation concerns the cut-off points chosen for the scores of the questionnaires used. The current study did not include a community sample of healthy controls, which prevented us from setting a locally adjusted cut-off point for the SRQ-20 or PSS. However, compared to other studies that used the SRQ-20 a conservative cut-off point was chosen. [61,62] The PSS had not been used as widely, as it is a simplified version of the original P-Scale and therefore uses different cut-off values. For the EMIC, no cut-off points can be determined through a healthy control group as not perceiving or experiencing stigma would lead to a score of zero.

### Conclusion and recommendations

This study shows that many persons affected by leprosy or LF in Northern Mozambique experience severe psychosocial consequences related to their disease and the associated disabilities. Given the stigma experienced by persons affected by leprosy or LF, and the consequences for their mental wellbeing and social participation, these challenges need to be taken up urgently by NTD programmes to promote the inclusion and wellbeing of persons affected by NTDs. This could be done through disease management, disability, and inclusion (DMDI) services. [31] In addition to primary and secondary prevention of disabilities this approach also comprises tertiary prevention that includes psychological and socioeconomic support for people with disabling conditions to ensure that they have equal access to rehabilitation services and opportunities for health, education, and income. It is recommended that follow-up research is done, which includes a representative sample of the study population, to make sure that all persons affected by these diseases who need it are targeted by the relevant interventions. In addition, it is important that further strategies are explored for contextualising mental health care approaches in self-care groups, trying to understand local models to explain and treat persons with mental distress, and to integrate with global mental health best practices.

Funding

This work was supported by the Coalition for Operational Research on Neglected Tropical Diseases (COR-NTD) [https://www.cor-ntd.org/], which is funded at The Task Force for Global Health primarily by the Bill & Melinda Gates Foundation, by UKAid from the British government, and by the United States Agency for International Development through its Neglected Tropical Diseases Program.” (NTD-SC #165D DATED January 1, 2019. P.O #2707). The funders had no role in study design, data collection and analysis, decision to publish, or preparation of the manuscript.

Competing interests

None declared.

Ethical approval

Ethical approval for this study was obtained on 12 September 2019 from the Comité Nacional de Bioética para Saúde (CNBS) in Mozambique, registration number: 32/CNBS/2019.

Data availability

The data that support the findings of this study are available via: https://www.infontd.org/sites/default/files/2024-01/240131%20LF-leprosy%20Moz%20quant%2Bqual%20data%20-%20publication%20InfoNTD.pdf

## References

1.	World Health Organisation. Ending the neglect to attain the Sustainable Development Goals: a road map for neglected tropical diseases 2021–2030. Geneva; 2020.

2.	Uniting to Combat NTDs. Mozambique and neglected tropical diseases - Mass Treatment Coverage for NTDs - 2016. 2018.

3.	World Health Organisation. Leprosy factsheet [Internet]. 2023 [cited 2023 Jan 2]. Available from: https://www.who.int/news-room/fact-sheets/detail/leprosy

4.	van Brakel WH, Sihombing B, Djarir H, Beise K, Kusumawardhani L, Yulihane R, et al. Disability in people affected by leprosy: the role of impairment, activity, social participation, stigma and discrimination. Glob Health Action. 2012;5.

5.	World Health Organisation. Lymphatic filariasis factsheet [Internet]. 2022 [cited 2023 Jan 2]. Available from: https://www.who.int/news-room/fact-sheets/detail/lymphatic-filariasis

6.	Douglass J, Kelly-Hope L. Comparison of staging systems to assess lymphedema caused by cancer therapies, lymphatic filariasis, and podoconiosis. Lymphat Res Biol. 2019;17(5):550–6.

7.	Grau-Pujol B, Massangaie M, Cano J, Maroto C, Ndeve A, Saute F, et al. Frequency and distribution of neglected tropical diseases in Mozambique: a systematic review. Infect Dis Poverty. 2019;8(06):1–11.

8.	Grau-Pujol B, Massangaie M, Cano J, Maroto C, Ndeve A, Saute F, et al. Frequency and distribution of neglected tropical diseases in Mozambique: a systematic review. Infect Dis Poverty. 2019;8(06):1–11.

9.	World Health Organization. https://apps.who.int/neglected_diseases/ntddata/leprosy/leprosy.html. 2023. Leprosy: number of new leprosy cases 2019.

10.	Ministério de Saúde Moçambique. News Report Deputy Minister of Health. 2021;

11.	World Health Organization. https://apps.who.int/neglected_diseases/ntddata/lf/lf.html. 2023. Lymphatic filariasis: status of mass drug administration 2021.

12.	Brouwers C, van Brakel W, Cornielje H. Quality of life, perceived stigma, activity and participation of people with leprosy-related disabilities in south-east Nepal. Disability, CBR & Inclusive Development. 2011;22(1):16–34.

13.	Hofstraat K, van Brakel WH. Social stigma towards neglected tropical diseases: a systematic review. Int Health [Internet]. 2016;8(suppl 1):i53–70. Available from: https://academic.oup.com/inthealth/article-lookup/doi/10.1093/inthealth/ihv071

14.	Pierneef L, van’t Noordende AT, Bista M, Singh U, Scheltema MB, Joshi AK, et al. Factors influencing the mental wellbeing of persons affected by leprosy in Far-Western Nepal. Lepr Rev. 2022;93(1):12–25.

15.	Somar PMW, Waltz MM, van Brakel WH. The impact of leprosy on the mental wellbeing of leprosy-affected persons and their family members–a systematic review. Global Mental Health. 2020;7:e15.

16.	Ali O, Mengiste A, Semrau M, Tesfaye A, Fekadu A, Davey G. The impact of podoconiosis, lymphatic filariasis, and leprosy on disability and mental well-being: A systematic review. PLoS Negl Trop Dis. 2021;15(7):e0009492.

17.	World Health Organization. Mental health of people with neglected tropical diseases: towards a person-centred approach. World Health Organization; 2020.

18.	Kuper H. Disability, mental health, stigma and discrimination and neglected tropical diseases. Trans R Soc Trop Med Hyg. 2021;115(2):145–6.

19.	Mieras LF, Anand S, van Brakel WH, Hamilton HC, Martin Kollmann KH, Mackenzie C, et al. Neglected Tropical Diseases, Cross-Cutting Issues Workshop, 4–6 February 2015, Utrecht, the Netherlands: meeting report. Int Health. 2016;8(suppl_1):i7–11.

20.	Gilman J, Myatt M. EpiCalc 2000. Version 1.02. Brixton Books. London, United Kingdom. 1998.

21.	World Health Organization. User’s guide to the Self Reporting Questionnaire (SRQ). 1994.

22.	Van Brakel WH, Anderson AM, Mutatkar RK, Bakirtzief Z, Nicholls PG, Raju MS, et al. The Participation Scale: Measuring a key concept in public health. Disabil Rehabil. 2006;28(4):193–203.

23.	Rensen C, Bandyopadhyay S, Gopal PK, Van Brakel WH. Measuring leprosy-related stigma–a pilot study to validate a toolkit of instruments. Disabil Rehabil. 2011;33(9):711–9.

24.	Vanderbilt. Project REDCap. 2020.

25.	Tsutsumi A, Izutsu T, Md Islam A, Maksuda AN, Kato H, Wakai S. The quality of life, mental health, and perceived stigma of leprosy patients in Bangladesh. Soc Sci Med. 2007;64(12):2443–53.

26.	Weiss MG, Doongaji DR, Siddhartha S, Wypij D, Pathare S, Bhatawdekar M, et al. The Explanatory Model Interview Catalogue (EMIC). Contribution to cross-cultural research methods from a study of leprosy and mental health. The British Journal of Psychiatry. 1992;160(6):819–30.

27.	Van Brakel WH. Measuring health-related stigma—a literature review. Psychol Health Med. 2006;11(3):307–34.

28.	International Federation of Anti-Leprosy Associations and Neglected Tropical Disease NGO Network. Guides on Stigma and Mental Wellbeing. [Internet]. 2020 [cited 2023 Jan 2]. Available from: stigmaguides.org

29.	Prevention C for DC and. Community health assessment aNd group evaluation (CHANGE) action guide: building a foundation of knowledge to prioritize community needs. Atlanta: US Department of Health and Human Services. 2010;

30.	Organization WH. Lymphatic filariasis: managing morbidity and preventing disability: an aide-mémoire for national programme managers. World Health Organization; 2013.

31.	NTD NGO Network. Disease Management, Disability and Inclusion [Internet]. 2019 [cited 2023 Jan 6]. Available from: disease management, disability, and inclusion (DMDI). This also represents tertiary prevention that includes psychological and socioeconomic support for people with disabling conditions to ensure that they have equal access to rehabilitation services and opportunities for health, education, and income.

32.	Cunha MADS, Antunes DE, Da Silveira RWM, Goulart IMB. Application of the SRQ20 and the protocol of psychological assessment in patients with leprosy in a Reference Centre in Brazil. Lepr Rev [Internet]. 2015;86(3):229–39. Available from: http://www.ncbi.nlm.nih.gov/pubmed/26665358

33.	Vos-Klootwijk L de. Cross-Cultural validation of two scales to assess stigma and social participation in leprosy-affected persons in Kathmandu Valley, Nepal. 2019.

34.	Sermrittirong S, Van Brakel WH, Kraipui N, Traithip S, Bunders-Aelen JFG. Comparing the perception of community members towards leprosy and tuberculosis stigmatisation. Lepr Rev. 2015;86(1):54–62.

35.	Alonso LM. Stigmatizing neglected tropical diseases: a systematic review. Social Medicine. 2010;5(4):218–27.

36.	Wilsher E. The impact of Neglected Tropical Diseases, and their associated stigma, on people’s basic capabilities. 2011.

37.	World Health Organisation. Leprosy NTD data [Internet]. 2020 [cited 2023 Jan 6]. Available from: https://apps.who.int/neglected_diseases/ntddata/leprosy/leprosy.html

38.	Marega A, Hambridge T, Stakteas YP, Schoenmakers A, van Wijk R, Mieras L, et al. Leprosy indicators and diagnosis delay in Mogovolas, Meconta and Murrupula district of Nampula Province, Mozambique: A baseline survey. Lepr Rev. 2022;93(3):197–208.

39.	Adekeye O, Chowdhury S, McRae A, Olorunfemi T, Ozokede E, Dubukumah L, et al. Exploring the well-being of people affected by skin NTDs in Kaduna and Kwara States, Nigeria: a photovoice and scoping review study. Int Health. 2023;15(Supplement_1):i100–9.

40.	van ‘t Noordende AT, Aycheh MW, Schippers A. The impact of leprosy, podoconiosis and lymphatic filariasis on family quality of life: A qualitative study in Northwest Ethiopia. PLoS Negl Trop Dis. 2020;14(3):e0008173.

41.	van Brakel WH, Peters RM, da Silva Pereira ZB. Stigma Related to Leprosy—A Scientific View. The International Textbook of Leprosy. 2019;1:1–32.

42.	Frota da Rocha Morgado F, Kopp Xavier da Silveira EM, Pinheiro Rodrigues do Nascimento L, Sales AM, da Costa Nery JA, Nunes Sarno E, et al. Psychometric assessment of the EMIC Stigma Scale for Brazilians affected by leprosy. PLoS One. 2020;15(9):e0239186.

43.	Adhikari B, Kaehler N, Chapman RS, Raut S, Roche P. Factors affecting perceived stigma in leprosy affected persons in western Nepal. PLoS Negl Trop Dis. 2014;8(6):e2940.

44.	Ebenso B, Newell J, Emmel N, Adeyemi G, Ola B. Changing stigmatisation of leprosy: an exploratory, qualitative life course study in Western Nigeria. BMJ Glob Health. 2019;4(2):e001250.

45.	Eneanya OA, Garske T, Donnelly CA. The social, physical and economic impact of lymphedema and hydrocele: a matched cross-sectional study in rural Nigeria. BMC Infect Dis. 2019;19(1):1–16.

46.	Abdulmalik J, Nwefoh E, Obindo J, Dakwak S, Ayobola M, Umaru J, et al. Emotional difficulties and experiences of stigma among persons with lymphatic filariasis in Plateau State, Nigeria. Health Hum Rights. 2018;20(1):27.

47.	van Dorst MMAR, van Netten WJ, Waltz MM, Pandey BD, Choudhary R, van Brakel WH. Depression and mental wellbeing in people affected by leprosy in southern Nepal. Glob Health Action. 2020;13(1):1815275.

48.	Pierneef L, van’t Noordende AT, Bista M, Singh U, Scheltema MB, Joshi AK, et al. Factors influencing the mental wellbeing of persons affected by leprosy in Far-Western Nepal. Lepr Rev. 2022;93(1):12–25.

49.	Van Netten WJ, Van Dorst MMAR, Waltz MM, Pandey BD, Aley D, Choudhary R, et al. Mental wellbeing among people affected by leprosy in the Terai region, Nepal. Lepr Rev. 2021;92(1):59–74.

50.	Obindo J, Abdulmalik J, Nwefoh E, Agbir M, Nwoga C, Armiya’u A, et al. Prevalence of depression and associated clinical and socio-demographic factors in people living with lymphatic filariasis in Plateau State, Nigeria. PLoS Negl Trop Dis. 2017;11(6):e0005567.

51.	Abdulmalik J, Nwefoh E, Obindo J, Dakwak S, Ayobola M, Umaru J, et al. Emotional difficulties and experiences of stigma among persons with lymphatic filariasis in Plateau State, Nigeria. Health Hum Rights. 2018;20(1):27.

52.	Lund C, De Silva M, Plagerson S, Cooper S, Chisholm D, Das J, et al. Poverty and mental disorders: breaking the cycle in low-income and middle-income countries. The lancet. 2011;378(9801):1502–14.

53.	Baumeister RF, Stillwell AM, Heatherton TF. Guilt: an interpersonal approach. Psychol Bull. 1994;115(2):243.

54.	Crocker J, Wolfe CT. Contingencies of self-worth. Psychol Rev. 2001;108(3):593.

55.	Matthews T, Danese A, Wertz J, Odgers CL, Ambler A, Moffitt TE, et al. Social isolation, loneliness and depression in young adulthood: a behavioural genetic analysis. Soc Psychiatry Psychiatr Epidemiol. 2016;51:339–48.

56.	Sharaf AY, Ossman LH, Lachine OA. A cross-sectional study of the relationships between illness insight, internalized stigma, and suicide risk in individuals with schizophrenia. Int J Nurs Stud. 2012;49(12):1512–20.

57.	Oexle N, Rüsch N, Viering S, Wyss C, Seifritz E, Xu Z, et al. Self-stigma and suicidality: a longitudinal study. Eur Arch Psychiatry Clin Neurosci. 2017;267:359–61.

58.	Carpiniello B, Pinna F. The reciprocal relationship between suicidality and stigma. Front Psychiatry. 2017;35.

59.	Deepak S, Hansine PE, Braccini C. Self-care groups of leprosy-affected people in Mozambique. Lepr Rev. 2013;84(4):283–91.

60.	De Kruijff A. Mozambique Country Profile. Lepr Rev. 2015;86(1):89–95.

61.	Netsereab TB, Kifle MM, Tesfagiorgis RB, Habteab SG, Weldeabzgi YK, Tesfamariam OZ. Validation of the WHO self-reporting questionnaire-20 (SRQ-20) item in primary health care settings in Eritrea. Int J Ment Health Syst. 2018;12(1):1–9.

62.	Giang KB, Allebeck P, Kullgren G, Van Tuan N. The Vietnamese version of the Self Reporting Questionnaire 20 (SRQ-20) in detecting mental disorders in rural Vietnam: a validation study. International Journal of Social Psychiatry. 2006;52(2):175–84.
