# Supplementary material for: Leprosy and lymphatic filariasis-related disability and psychosocial burden in northern Mozambique
Source: PLoS Negl Trop Dis. 2024 Aug 13;18(8):e0012342. doi: 10.1371/journal.pntd.0012342 (PMC11321553; doi:10.1371/journal.pntd.0012342)
Supplement: Attachment — Submitted filename: 240531 Reviewer comments & responses.docx [file pntd.0012342.s002.docx]

## Dear reviewers,

Many thanks for the time and effort that you have put in reviewing our manuscript.

We greatly appreciate your comments for improvement and we have addressed them carefully.

Kindly find below an overview of your comments and our response. We make reference to the lines in the manuscript where you can find the adjustments. These are also highlighted in the track changes version of the manuscript.

Best wishes,

On behalf of all authors

Robin van Wijk

## Reviewers’ comments and authors’ responses

**Abstract**

| Reviewer comment | Response |
| --- | --- |
| 24: write emic in full | Adjusted in the text line 25. |
| Line 24: add number of interviews | The number of interviews conducted (eight) is mentioned in the results section of the abstract (line 30), this structure corresponds with the structure of the full text. |
| Result- line 29-30: More severe disabilities were associated with higher scores for all questionnaires. You have to specify the term questionnaires, to make it clear for the readers. it has to be replaced with the outcomes you want to indicate. | Thank you for pointing this out. The outcomes are now mentioned instead of “questionnaires” – line 35-36. |
| 32: showed = was demonstrated | Adjusted in the text line 37. |
| Author summary: add 127 persons affected by leprosy and 184 persons affected by LF were included in the quantitative study component and eight semi-structured interviews were conducted. | Adjusted in the text line 51-52. |

**Introduction**

| Reviewer comments | Response |
| --- | --- |
| Is lymphedema pitting? Confirm | Lymphedema can be pitting or non-pitting oedema (swelling). Pitting oedema is when a mark is left in the skin after applying pressure from a finger, hand, or object. |
| I would suggest some rewriting of the sentences around 'irreversible impairments' to make it clear that level of ongoing impairments is what matters, but not all symptoms are irreversible. | Thank you for pointing this out. This information has been added to the text, line 97-98. |

**Methods**

| Reviewer comments | Response |
| --- | --- |
| Say a community based cross-sectional mixed-methods study | Adjusted, line 120. |
| What to mean by a person affected by leprosy or LF? You have to give clear operational definition for “a person affected by leprosy or LF”. A person affected by leprosy or LF can be different from a person infected by leprosy or LF. Your outcomes like psychosocial burden is the common problem of infected individuals and their family members and/or primary caregivers. Therefore, I want to be clear with your study population. | Thank you for pointing this out. The definition has been added to the text, line 146-149. |
| Has this instrument been validated and used in Mozambique or any African Country. What was the suggested cut-off point for mental distress? | The SRQ-20 was validated in Ethiopia, Rwanda and Zambia – this was added to the text, line 193-194. The used cut-off is mentioned under the heading “data analysis”, line 269. |
| Sample size for qualitative study is not clearly stated. | Thank you for identifying this, elaboration on the sample size has been added to the text, line 227-230. |
| Data Analysis line 267 Multivariable analysis needs more detail as to what models were used and precise description of the outcomes/ dependent variables. | Thank you for pointing this out. The text has been revised, line 288-293. |
| Why you excluded the study participants if they were affected by more than one of the target diseases (leprosy and LF)? For instance, why you didn’t try to see psychosocial burden among those who are affected by both leprosy and LF compared to those who affected by one of them? | Interesting question and proposal. We decided that if we included those affected by both diseases it would not be possible to differentiate between the impact of each disease. It would indeed be interesting to compare those affected by 2 diseases to those affected by 1, but that would require a high number of participants with both diseases to find statistically significant results. |
| Clearly elaborate what type of data you collected by using quantitative survey and using interview respectively | This comment is not clear, since the respective data collection tools are already described in some detail. In the results text we added when quantitative and when qualitative results were described. |
| 10% is often added to the calculated sample size to give room for attritions and poorly filled questionnaires. | Thank you for your advice |

**Results**

| Reviewer comments | Response |
| --- | --- |
| Add a sub-heading for qualitative part of the study. | The authors made the choice to integrate the qualitative information with the quantitative results. Using sub headings would lead to many sub headings and would decrease readability. In the text it was added when quantitative and when qualitative results were described. |
| Line 315:– Write in full DPO | DPO was written in full on its first use in the methods section, line 228. |
| 322: rewrite sentence ADEMO is a DPO active in several district of Nampula, among which the studied districts. | Rewritten, line 228-230. |
| Line 326, says, Grade 1 Grade 2. May need edition. | Thank you, the words have been deleted. |
| What to mean by “7% it was not graded”? Is it to mean 0 grade? | It means that the lymphoedema grade was unknown because it was not recorded, this was adjusted in the text, line 353-354. |
| Line 361. Why do Fig 3 &4 get mentioned before Fig 2. They should be numbered in the order that they are presented in the text. The figs with the individual domains should all be presented first before the combined graph of the composite scores | Thank you for noticing this. We have adjusted the text to correspond with the order of the figures, line 390-393. |
| Table 6 needs more explanation in the caption. | Adjusted, line 496 |
| Table 6. Why you prefer linear regression analysis for categorical outcomes? Why not binary logistic regression? | A multivariate analysis was carried out using linear regression models with mental distress, participation restriction and stigma as the continuous dependent outcomes. Collapsing these to binary outcome variables would have reduced the power to find associations substantially. |

**Discussion**

| 502-508: This is more like “limitation to the study” rather than discussion of the result! | This was also mentioned in the limitations section. However, at this point in the discussion it is mentioned as a comparison to the general population, but still highlighting the importance of the impact of impairment on mental distress, participation restriction and stigma. |
| --- | --- |

**References**

| Reviewer comments | Response |
| --- | --- |
| References comments regarding duplication and mentioning of date of access for webpages. | Thank you, these have been taken up. |
